# Advantages of routine next‐generation sequencing over standard genetic testing in the amyotrophic lateral sclerosis clinic

**DOI:** 10.1111/ene.15855

**Published:** 2023-05-28

**Authors:** Jakub Scaber, Alexander G. Thompson, Lucy Farrimond, Emily Feneberg, Malcolm Proudfoot, Lynn Ossher, Martin R. Turner, Kevin Talbot

**Affiliations:** ^1^ Nuffield Department of Clinical Neurosciences University of Oxford, John Radcliffe Hospital Oxford UK; ^2^ Kavli Institute for Nanoscience Discovery University of Oxford Oxford UK

**Keywords:** amyotrophic lateral sclerosis, genetics, whole exome sequencing

## Abstract

**Background:**

Next‐generation sequencing has enhanced our understanding of amyotrophic lateral sclerosis (ALS) and its genetic epidemiology. Outside the research setting, testing is often restricted to those who report a family history. The aim of this study was to explore the added benefit of offering routine genetic testing to all patients in a regional ALS centre.

**Methods:**

*C9ORF72* expansion testing and exome sequencing was offered to consecutive patients (150 with ALS and 12 with primary lateral sclerosis [PLS]) attending the Oxford Motor Neuron Disease Clinic within a defined time period.

**Results:**

A total of 17 (11.3%) highly penetrant pathogenic variants in *C9ORF72*, *SOD1*, *TARDBP*, *FUS* and *TBK1* were detected, of which 10 were also found through standard clinical genetic testing pathways. The systematic approach resulted in five additional diagnoses of a *C9ORF72* expansion (number needed to test [NNT] = 28), and two further missense variants in *TARDBP* and *SOD1* (NNT = 69). Additionally, 3 patients were found to carry pathogenic risk variants in *NEK1*, and 13 patients harboured common missense variants in *CFAP410* and *KIF5A*, also associated with an increased risk of ALS. We report two novel non‐coding loss‐of‐function splice variants in *TBK1* and *OPTN*. No relevant variants were found in the PLS patients. Patients were offered double‐blinded participation, but >80% requested disclosure of the results.

**Conclusions:**

This study provides evidence that expanding genetic testing to all patients with a clinical diagnosis of ALS enhances the potential for recruitment to clinical trials, but will have direct resource implications for genetic counselling.

## INTRODUCTION

Amyotrophic lateral sclerosis (ALS) is characterised by progressive neurodegeneration of the corticospinal tract and alpha motor neurons in the spinal cord. There is a clinical, histopathological and genetic overlap with frontotemporal dementia (FTD). The mechanisms underlying this neurodegeneration, its cellular specificity, and its relationship to ageing remain incompletely understood [1].

The importance of genetic factors in the pathophysiology of ALS has been long known in the clinic [2]. Between 2% and 12% [3] of patients report a relevant family history of the disease, with estimates diverging widely due to differences in study populations, distortions in ascertainment due to referral bias, and lack of consensus definitions of what constitutes familial ALS [4]. Autosomal dominant variants with high penetrance in *C9ORF72*, *SOD1*, *TARDBP* and *FUS* account for more than half of all cases with a family history but, importantly, are also be found in patients without affected family members [5]. Even so, these variants only account for a proportion of the total heritability, which has been estimated at around 50% in twin studies [6] and population studies [7]. Over the past decade, high‐throughput sequencing technologies and genome‐wide association studies (GWAS) have contributed to the elucidation of the genetic architecture of ALS. This has led to the identification of further rare monogenic causes [8], and also the discovery of rare risk variants that do not segregate with disease, but which are found significantly more frequently in ALS patients than in controls such as *NEK1* [9], or common variants which modulate the severity and onset of disease such as *UNC13A* [10].

Targeted high‐throughput exome sequencing, now available in the form of ‘virtual sequencing panels’, allows simultaneous testing of all known genes associated with ALS at a cost similar to Sanger sequencing of a single gene. Such panels have become a routine tool in clinics specialising in hereditary neurological disorders [11], but their utility in the ALS clinic remains under investigation, and testing is usually available only on a research basis for patients without a family history. Previous studies using ALS genetic panels have reported ‘pathogenic’ and ‘likely pathogenic’ variants in 12%–21% overall in clinic populations [12, 13, 14, 15, 16, 17], with a detection rate ranging from 5% to 13% in patients who do not report a family history.

This aim of this study was to investigate the genetic contribution to ALS in an unselected clinic population in a large UK ALS referral centre and to understand the clinical utility of offering *C9ORF72* testing and targeted exome sequencing to all patients, in addition to standard approaches to genetic testing. A systematic analysis of known, previously reported ALS variants was extended by using codon‐based analysis to look for neighbouring ALS‐associated variants and machine‐learning algorithms to look for splice site variation, yielding novel deleterious splice donor variants in *TBK1* (c.1340 + 2 T > G) and in *OPTN* (c.1242 + 1G > A).

## METHODS

This study was carried out under ethical approval from the local Oxford Research Ethics Committee (REC Ref/ CTA No: 15/SC/0469). To maintain anonymity, results were reported back to a ‘genetic guardian’ and, where the subject had selected non‐disclosure, the results were communicated only as part of the description of the data at group level, to avoid accidental unblinding of the clinical team.

During a 20‐month study period between 2016 and 2018, all patients attending the Oxford Motor Neuron Disorders Clinic with a relevant clinical diagnosis were offered genetic testing, regardless of their family history and duration of previous follow‐up. Genetic testing was undertaken in a research laboratory setting, involving repeat prime polymerase chain reaction (PCR) testing and Southern blotting for *C9ORF72* followed by whole exome sequencing with selective analysis of known or potential ALS genes. Participants provided informed consent for testing and were given the option to receive their results. The study team remained blinded to individual results for those not wishing to have feedback. All subjects received routine clinical care in addition to this study, which included standard investigation of patients who reported a relevant family history through routine clinical genetic testing pathways. Samples were taken from 163 unselected patients diagnosed with ALS, ALS with FTD, or primary lateral sclerosis (PLS) in a regional specialist ALS clinic. We performed a subgroup analysis restricted to patients tested within 2 years from first onset of symptoms, to provide an approximation of an incident population (*n* = 65; Table 1).

**TABLE 1:** Patient demographics.

Parameter	ALS (*n* = 150)	ALS <2 years from onset (*n* = 65)	PLS (*n* = 12)
Age at onset, median (IQR), years	61 (52–67)	65 (53–72)	53 (50–63)
Median survival (95% CI), years	5.0 (4.1–6.5)	2.8 (2.6–3.2)	19.4 (〉19.2)
10‐year survival (95% CI)	27% (19%–37%)	–	90% (76%–100%)
Onset	21% bulbar 37% upper limb 35% lower limb		25% bulbar 75% lower limb

Abbreviations: ALS, amyotrophic lateral sclerosis; CI, confidence interval; IQR, interquartile range; PLS, primary lateral sclerosis.

Exome sequencing was carried out at University College London (Prof. P. Fratta) on the Illumina HiSeq platform following library preparation with Agilent SureSelect v6 post‐capture chemistry. Mapping to the reference genome hg38 was performed using Bwa‐mem2 (v. 2.2.1) [18]. Variant analysis was performed in accordance with the Broad Institute GATK (Genome Analysis Toolkit) best practices for germline short variant discovery, using HaplotypeCaller in GVCF mode and joint calling of the entire cohort using GenotypeGVCFs (v. 4.2.6.1) [19]. Quality filtering was performed using the GATK variant quality score recalibration algorithm. Variant annotation was performed using Variant Effect Predictor (VEP, option ‐e, v105GRCh38) [20], which was run with optional plugins SameCodon, SpliceAI (database v1.3) [21] and dbNSFP (database v4.2a, option ALL) [22].

Variant calling was restricted to genes where there is consensus about a causal link to ALS (*ANXA11*, *CHCHD10*, *EPHA4*, *FUS*, *HNRNPA1*, *KIF5A*, *NEK1*, *OPTN*, *PFN1*, *SOD1*, *TARDBP*, *TBK1*, *UBQLN2*, *VAPB*, *VCP*) or where there is strong evidence for an association with ALS (*CCNF*, *CFAP410*, *HFE*, *NIPA1*, *SCFD1*, *TUBA4A*), as classified by the Amyotrophic Lateral Sclerosis Online Database (ALSoD) [23]. Variants were filtered using a browser extensible data file, which was generated from an Ensembl version 101 gene transfer format file. As expected for exome sequencing, there was no coverage for known intronic *UNC13A* single nucleotide polymorphisms, and this definitively ALS‐associated gene was therefore not included in the analysis.

Variant filtering was performed using the Ensembl Variant Effect Predictor (VEP), with filter_vep options as outlined below:
 ((Consequence != synonymous_variant) and ((clinvar_trait match Amyotrophic) or (clinvar_trait match Motor_neuron) or (SameCodon match [list of all ALS‐related SNVs]))) or (SpliceAI_pred_DS_AG >0.5 or SpliceAI_pred_DS_AL >0.5 or SpliceAI_pred_DS_DG >0.5 or SpliceAI_pred_DS_DL >0.5)



To check for variants not reported on ClinVar, all variants with ExaC minor allele frequency <5% were checked against a curated list of previously published ALS mutations [24].

Manual ascertainment of the variants with the lowest coverage and quality was performed on the final list, resulting in the rejection of one variant. In silico analysis was performed using MutationTaster [25], PolyPhen2 [26], SIFT [27] and FATHMM [28]. Filtered variants were interpreted using the standards and guidelines for the interpretation of sequence variants by the American College of Medical Genetics and Genomics (ACMG) [29].

Relatedness of samples and ancestry were evaluated using Somalier [30].

Statistical analyses, including Kaplan–Meier survival analysis (census date 1 July 2022), were performed in R (v. 4.1.2) and Graphpad Prism (v. 9.3.1) was used to generate illustrations.

## RESULTS

### Pathogenic variants

Consent for genetic testing was obtained from 163 subjects, 148 of whom had a diagnosis of ALS, 3 ALS/FTD and 12 PLS. DNA extraction failed in one individual with ALS. The demographic data of the participants is outlined in Table 1. In four patients a *C9ORF72* result was reported but no sequencing panel data was generated, and in four patients a sequencing panel result was available, but extraction of sufficient DNA for Southern blotting failed. Some 132/163 patients (81%) expressed a wish to be informed of any genetic results relevant to them arising from the study. All but eight participants were of European ancestry using a computational estimate, and none of the participants were found to be interrelated.

**FIGURE 1:**
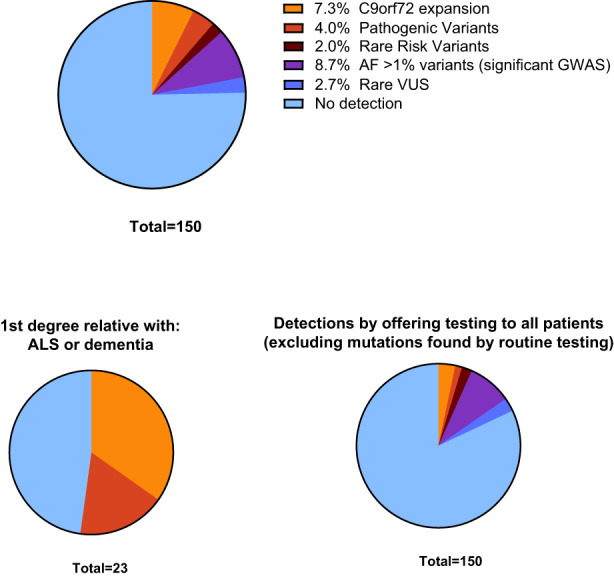
Pie charts showing the frequencies of variants found in this study. For patients with co‐occurring variants (see Appendix), only the more pathogenic variant is shown. Upper panel shows findings in all amyotrophic lateral sclerosis (ALS) patients (excluding primary lateral sclerosis). Lower panels show the frequency of positive findings in patients with a family history (left) and variants that were not detected by the clinical genetics service (right). AF, allele frequency; GWAS, genome‐wide association study; VUS, variant of unknown significance.

In the 150 patients with ALS, 16 pathogenic and 1 likely pathogenic ALS‐causing variants were detected in our cohort according to ACMG criteria (11.3%), rising to 12/23 (48%) for ALS patients who reported a family history of ALS or dementia in a first‐degree relative (Figure 1). In the subset of 65 incident patients with disease onset within 2 years of their genetic test, 8 pathogenic ALS‐causing variants were detected (12.3%).

The *C9ORF72* hexanucleotide expansion was the most common highly penetrant pathogenic variant found in this study, detected in 11/150 (7.3%) patients. Of the 10 patients whose identity is known, four reported a family history of ALS in a first‐degree relative, two reported a family history of dementia in a first‐degree relative, one reported a first‐degree relative with multiple sclerosis, and four patients reported no family history of neurological disease. The age of onset in *C9ORF72*‐related ALS ranged from 42 to 67 years, with limb onset in eight patients, and one each with cognitive or bulbar onset. In five patients the *C9ORF72* expansion was only found through the systematic screening approach, as patients were not selected for routine testing.

The analysis of pathogenic and rare variants is summarised in Table 2. In three patients who reported a relevant family history in a first‐degree relative, routine clinical testing had already identified pathogenic variants in *SOD1* (I114T and H47R) and a likely pathogenic variant in *FUS* (R514S). In addition to this, the research sequencing panel detected a further *SOD1* I114T and a *TARDBP* A315T variant in patients who did not report a family history.

**TABLE 2:** Pathogenic and rare variants found in the current study.

Cases	Gene	Position	SNP	Protein change	Published in ALS	GnomAD frequency (non‐Finnish European)	Predictive data	Functional data	Segregation	ACMG interpretation
LO ALS, typical progression (2 patients)	*SOD1*	c.341 T > C Located in hotspot (PM1)	rs121912441	I114T	Commonest pathogenic *SOD1* variant in UK population, extensive evidence (PP5) [45]	Absent (PM2)	Multiple Deleterious (PP3)	Supportive: SOD1‐positive pathology [46]	Yes: extensive co‐segregation in cohorts from different continents [45, 47]	Pathogenic (1 strong, 2 moderate, 2 supporting)
LO ALS, <50 years, slowly progressive	*SOD1*	c.140A > G Located in hotspot (PM1)	rs121912443	H47R	Extensive publications; clinical phenotype concordant – commonly slowly progressive.	Absent (PM2)	Deleterious (PP3)	No relevant functional studies	Yes: extensive co‐segregation in cohorts from different continents [48, 49]	Pathogenic (1 strong, 2 moderate, 2 supporting)
LO ALS, typical progression	*TARDBP*	c.943G > A	rs80356726	A315T	Extensive publications	0.0009%	Deleterious (PP3)	No relevant functional studies	Yes [50]	Pathogenic (1 strong, 2 moderate, 2 supporting)
LO ALS, typical progression	*FUS*	c.1542G > T Located in hotspot (PM1)	rs1555509681	R514S	Yes, multiple ethnicities [51, 52]	Absent (PM2)	Deleterious (PP3)	Supportive: cellular mislocalisation [52]	Observed in familial ALS, but co‐segregation not assessed	Likely pathogenic (2 moderate, 2 supporting)
ALS, onset <50 years, typical progression	*TBK1*	c.1340 + 2 T > G	Not in variant database	A417X	Adjacent to c.1340 + 1G > A, which is predicted to cause premature stop codon [31]	Absent (PM2)	LOF (PVS1)	Supportive: c.1340 + 1G > A causes haploinsufficiency (PS3)	Yes: cosegregation of LOF variants observed in multiple European pedigrees [31] (PM2)	Pathogenic (1 very strong, 1 strong, 2 moderate)
LO ALS, slow progression (2 patients)	*NEK1*	c.3107C > G	rs19994719	S1036X	OR 8.8 in large case–control study in sALS and fALS [9] (PS4)	Absent	LOF (PVS1)	No relevant functional studies	Limited evidence of non‐segregation/reduced penetrance [33] (BS4)	Pathogenic risk variant (1 very strong, 1 strong)
LO ALS, slow progression	*NEK1*	c.782G > A	rs200161705	R261H	OR 2.4 in large case–control study in sALS and fALS [9] (PS4)	0.4%	Deleterious (PP3)	No relevant functional studies	Not reported	Pathogenic risk variant (1 strong, 1 supportive)
LO ALS, slow progression	*OPTN*	c.1242 + 1G > A	rs120647814	E414X	c. 1242 + 1G > A_insA variant previously reported [32]	Absent (PM2)	LOF (PSV1)	No relevant functional studies	Not reported	VUS – as heterozygous
LO ALS, slow progression	*SOD1*	c.272A > C Located in hotspot	rs80265967	D91A	Extensive publications, slowly progressive phenotype for homozygotes [53]	0.1% (all heterozygous)	No consensus	No SOD1 pathology in heterozygotes [37]	Yes: extensive recessive pedigrees	VUS – as heterozygous
BO ALS, typical progression	*ANXA11*	c.112G > A	rs142083484	G38R	UK: 2 [35] France: 2 [34] S.Korea: 1 [36] Germany: 1 [54] (PP5)	0.007%	Deleterious (PP3)	Supportive: enhances aggregation propensity [36] ANXA11‐positive inclusions at post‐mortem [34]	Not reported	VUS
ALS, onset <50 years, typical progression	*TBK1*	c.452C > T	rs55824172	S151F	UK: 1 [12] Also reported in glaucoma: 2 [55]	0.0009%	Deleterious (PP3)	No relevant functional studies	Not reported	VUS
PLS	*PFN1*	g.4945973 T > C	rs140547520	E117G	USA: 3 [56] but also in 3 controls *PFN1* cases have flaccid paresis	0.1%	Deleterious (PP3)	No relevant functional studies	Not reported	VUS

*Note*: Table ordered by likelihood of pathogenicity, according to ACMG guidance criteria [29]. References for Table 2 in Supplementary Material.

Abbreviations: ACMG, American College of Medical Genetics and Genomics; ALS, amyotrophic lateral sclerosis; BO, bulbar onset; fALS, familial ALS; sALS, sporadic ALS; FTD, frontotemporal dementia; LO, limb onset; LOF, loss‐of‐function; OD, odds ratio; PLS, primary lateral sclerosis; VUS, variant of unknown significance.

The use of the machine‐learning prediction tool SpliceAI additionally identified two non‐coding variants predicted to influence splicing. The first of these was a pathogenic variant in a splice site of *TBK1* (c.1340 + 2 T > G) in a patient from a multigenerational family that included two first‐degree relatives with ALS. This variant has previously not been reported in the literature and is absent from gnomAD. Segregation analysis could not be performed in our case, but the novel *TBK1* variant affects the same canonical splice donor site to the reported pathogenic loss of function variant c.1340 + 1G > A (A417X), which has previously been confirmed to cause haploinsufficiency [31]. We also detected a splice variant in *OPTN* c.1242 + 1G > A similar to a previous c.1242 + 1G > AinsA variant in the same gene [32]. As this variant was heterozygous in our patient, its relevance is of uncertain significance.

### Rare risk variants and variants of unknown significance

The research panel also identified three variants in *NEK1*, all in patients who did not report a family history of ALS, dementia or other neurological disease, all of whom had slow disease progression, as defined by survival of more than 5 years (Table 2). These variants have previously been shown to be associated with an increased risk of ALS in a large cohort of >1000 familial and >2000 sporadic ALS patients [9], while not showing segregation in pedigrees in another study [33]. Two patients in our study carried the S1036X variant, which has previously been found in 1% of sporadic ALS patients but only 0.2% of controls (odds ratio [OR] 5.9) [9]. The third patient in the current study was found to carry the R261H variant in *NEK1*, which was previously reported in 1.6% of sporadic ALS patients and 0.7% of controls (OR 2.4) [9].

In addition to the pathogenic variants described above, this study also identified a further four rare variants of unknown significance in panel genes. This included an *ANXA11* G38R variant in a patient who did not report a family history of ALS or dementia, which has been previously described in three independent ALS cohorts [34, 35, 36]. Although segregation has not been shown in pedigrees to date, and its penetrance is unknown, neuropathological inclusions staining positive for Anxa11 protein have been demonstrated with this variant [34]. According to our interpretation of ACMG criteria, evidence for this variant is currently insufficient to categorise it as ‘likely pathogenic’. We also identified a heterozygous *SOD1* D91A variant which was classified as a variant of unknown significance, as its accepted mode of inheritance is recessive [37].

### Common variants associated with ALS

Multiple variants in ALS‐associated genes with minor allele frequencies >1% in the general population have been reported at higher frequencies in ALS cohorts than in the general population, frequently co‐occurring with other variants in the same individual [24]. We found five such previously reported variants in our study, namely *CFAP410* V58L, *KIF5A* P986L, *TBK1* V464A, *CCNF* V714M and *OPTN* M98K. Apart from the M98K variant, all other variants were observed in our cohort at frequencies between two‐ and four‐fold higher than those reported in gnomAD for healthy non‐Finnish Europeans. All variants are missense but are predicted to be tolerated using in silico prediction tools. Variants were stratified by the strength of evidence in previous genetic studies (Table 3), and only the two variants with strong evidence for association with ALS from GWAS were reported in the results figure (Figure 1). Finally, we tested the hypothesis that the distribution of all 62 variants reported in ALS patients in this study was enriched for digenic inheritance. The nine examples of digenic variants in this study (Table S1) matches the number of expected digenic variants in a random draw model with replacement (*p* > 0.05).

**TABLE 3:** Previously reported common (allele frequency [AF] >1% in GnomAD) amyotrophic lateral sclerosis‐associated variants found in the current study.

Gene	Position	SNP	Protein change	ALS cases (*n*)	PLS cases (*n*)	GnomAD frequency (non‐Finnish European)	ClinVar submissions	Evidence for association with ALS
*CFAP410*	c.172G > T	rs75087725	V58L	9/150 (6%)	1/15	1.1%	Unspecified: Benign	Strong: significant GWAS SNP [39, 57], missense variant; in silico predictions benign
*KIF5A*	c.2957C > T	rs113247976	P986L	6/150 (4%)	None	1.5%	Spastic paraplegia: VUS	Strong: significant GWAS SNP [39], missense variant; in silico predictions benign
*TBK1*	c.1391 T > C	rs35635889	V464A	12/150 (8%)	1/15	2%	ALS: benign	Weak: heterozygous LOF mutations in *TBK1* have best evidence base in ALS, significance of missense mutations unclear. V464A variant located in scaffold dimerisation domain, whereas functional missense variants more likely to be in kinase domain [58]
*CCNF*	c.2140G > A	rs61755288	V714M	8/150 (5.3%)	1/15	1.5%	Unspecified: Benign	Weak: reported in Appendix of original publication [59], subsequently in a large Australian population study [24]; no statistical analysis; in silico predictions benign
*OPTN*	c.293 T > A	rs11258194	M98K	6/150 (4%)	1/15	2.8%	ALS: Benign Glaucoma: pathogenic	Weak: homozygous LOF of *OPTN*. have best evidence in ALS. Common missense variant reported in German, Australian and UK cohorts but no statistical analysis or significance; in silico predictions benign

*Note*: All mutations were heterozygous. References for Table 3 in Supplementary Material.

Abbreviations: AF, allele frequency; ALS, amyotrophic lateral sclerosis; GWAS, genome‐wide association study; LOF, loss‐of‐function; PLS, primary lateral sclerosis; SNP, single nucleotide polymorphism; VUS, variant of unknown significance.

### Primary lateral sclerosis

No pathogenic or likely pathogenic ALS‐linked variants were detected in the 12 patients who carried a definite diagnosis of PLS, with no evidence of lower motor neuron signs or symptoms more than 4 years from disease onset [38]. One patient with PLS in this cohort had a rare variant of unknown significance in PFN1 that had previously been reported in patients with lower motor predominant ALS, but which was also reported in control subjects. Interestingly, the common variants (allele frequency >1%) in *CFAP410* V58L, *TBK1* V464A, *CCNF* V714M and *OPTN* M98K were also found in one PLS patient each (Table 3).

## DISCUSSION

The rapid translation of high‐throughput sequencing methods into clinical practice creates opportunities and challenges for a disorder with a complex genetic architecture such as ALS, in which the distinction between familial and sporadic disease is not absolute. The emergence of clinical trials of antisense oligonucleotides and other genetic therapies suggests that every patient with ALS should have access to genetic testing. However, the application of routine testing in a clinical setting requires both appropriate clinical expertise and bioinformatic resources which are not universally available.

To understand the potential impact of routine high throughput genetic screening in the ALS clinic we compared it with standard clinical genetic testing pathways. We identified a frequency of pathogenic and likely pathogenic highly penetrant ALS variants of 11.3%, with a similar frequency of 12.3% in an approximated incident subset. In contrast, routine clinical testing identified 10 of the 17 pathogenic or likely pathogenic variants. Of the seven patients identified only through this study, five were found through *C9ORF72* screening (number needed to test = 28), and only two additional pathogenic variants with clear implications for management and trial eligibility through the exome sequencing panel (number needed to test = 69), in contrast to a recent UK study in which more pathogenic variants were detected [13]. However, our study confirms that the application of routine genetic testing, at least for *C9ORF72* and *SOD1*, would significantly increase the pool of subjects available for trials of genetic therapies.

The lower frequency of monogenic ALS compared to some recent studies has a number of possible explanations. All patients who were under follow‐up in the recruiting clinic during the study period were eligible for participation, possibly enriching for slower progression and atypical ALS with a longer mean survival compared to a pure incident population. A subset analysis of cases tested within 2 years of symptom onset, however, yielded a comparable frequency, which compares well with reported frequencies in a large prospective Italian study restricted to incident cases [14], and other European and Asian population and clinic studies [5], arguing against a strong effect of case selection in our data, especially as PLS cases were analysed separately. Referrals to our regional centre almost exclusively come from a defined geographic area with a systematic referral pattern which reflects ALS as it presents to general neurologists in our region.

An important technical aspect of this study was the use of machine‐learning splice prediction algorithms, which allow for the detection of non‐coding variants even in exome sequencing data [21]. This approach is particularly valuable given the association of ALS with loss of function and splice‐site variants in *TBK1*, *OPTN*, *KIF5A* and *NEK1* among others, and enabled us to discover two splice donor variants not previously reported in ClinVar or the existing literature. Codon‐centric bioinformatic approaches also helped with identification of variants adjacent to previously reported variation.

In addition to highly penetrant monogenic variants, we also found multiple variants that are associated with ALS risk. The two *NEK1* variants found in three patients in this study have been well characterised previously, with an estimate of relative risk calculated [9]. We also report five common missense variants that have an allele frequency of >1% in the general population, which are present in our cohort at several‐fold frequency. These are at most weak risk variants, with varying evidence of association with ALS, the strongest being for *CFAP410* V58L and *KIF5A* P986L, both of which are significant GWAS hits, with the potential to be direct effectors of increased ALS risk [39]. The remaining variants are less well characterised beyond overrepresentation in ALS cohorts, and their overall significance remains unknown (Table 3). The example of *NEK1*, where both loss of function and the relatively common R261H missense variant are associated with ALS, albeit with varying strength [9], indicate that certain missense mutations in *TBK1* and *OPTN* could play a role in ALS, but evidence for this is currently insufficient. Finally, the fact that four of the five common missense variants found in our ALS patients were also detected in in our PLS cohort is noteworthy; but given the small sample size, further studies would be required to explore the possibility of convergence of some of these risk variants in PLS and ALS. Due to the limitations of whole exome sequencing and the study design we were unable to assess the prevalence of the intermediate ATXN2 CAG expansion, which has also been associated with ALS risk, in our cohort [40]. Given that there is an ongoing phase 1/2 trial evaluating the safety and tolerability of the antisense oligonucleotide BIIB105 which targets ATXN2 mRNA for degradation (NCT04494256) in patients with and without the intermediate expansion, we would recommend including testing for the ATXN2 repeat in future genetic studies.

In this study we chose to only include genes with the strongest evidence for an ALS association, diverging from commonly available ALS gene panels [13, 17]. In agreement with the ALSoD database we did not include genes associated with ALS in early studies that have not been replicated, such as *SQSTM1*, nor did we include genes that are implicated in a common alternative motor system disease, such as *SPG11* and *ALS2* [23]. We argue that these genes contain no definitely pathogenic variants relevant to ALS and do not have sufficient evidence for an association with ALS, which may explain the slightly lower frequency of variants of unknown significance in our study compared to some previous studies [13, 24]. A cohort study cannot provide evidence for the pathogenicity of new variants, with very few exceptions such as inactivating variants in genes with an established loss‐of‐function pathogenic mechanism. We have therefore adopted a conservative variant filtering strategy, and only reported missense variants with existing evidence for pathogenicity in ALS. We strongly caution against overreporting of variants of unknown significance in both research studies and clinical practice, given the significant resource implications and risks to personal wellbeing [41].

Genetic reports from ALS exome and genome panels will inevitably increase in complexity. While the number of highly penetrant variants with clearly actionable consequences for genetic counselling and eligibility for antisense trials that can be found with systematic genetic testing has not changed [5], the number of risk variants with evidence of association with ALS is increasing rapidly. Clinicians therefore need to be equipped to provide appropriate counselling about the high likelihood of finding a variant, when the risk of transmission is difficult to quantify, and where there is currently no direct impact on clinical management [42, 43]. With the advent of genetic therapies, clinicians will also need to understand the varying levels of evidence for variants in known ALS‐associated genes such as *SOD1* and be able to counsel patients on the likelihood of therapeutic success, taking into account all available evidence. The heterozygous *SOD1* D91A mutation reported in this study is illustrative of this, with a recent neuropathological assessment of the patient showing TDP‐43 instead of the expected SOD1 pathology [37].

From a research perspective, understanding the complex genetic architecture of ALS is of paramount importance to our understanding of the pathophysiology of the disease and will be increasingly important in trial stratification. There was a high level of consent to be informed of individual results in this study (81%). We strongly recommend the approach of offering double‐blinded enrolment, with clear safeguards that prevent unwanted disclosure of a blinded genetic result. The specialist ALS clinic is the ideal setting for a patient to have a meaningful discussion about genetic testing and its results, and continuing expansion of research testing is an important part of the pursuit of personalised approaches to therapy in ALS [1, 44].

## AUTHOR CONTRIBUTIONS

**Jakub Scaber:** Writing – original draft; formal analysis; software; data curation; visualization; investigation; methodology. **Lucy Farrimond:** Investigation. **Emily Feneberg:** Investigation. **Malcolm Proudfoot:** Project administration; conceptualization. **Lynn Ossher:** Project administration. **Martin R Turner:** Supervision; resources; funding acquisition; conceptualization. **Kevin Talbot:** Supervision; resources; funding acquisition; conceptualization.

## CONFLICT OF INTEREST STATEMENT

The authors declare that they have no competing interests.

## Supporting information


Table S1.

## Data Availability

The data that support the findings of this study are available on request from the corresponding author. The data are not publicly available due to privacy or ethical restrictions.
