# Supplementary material for: Advantages of routine next‐generation sequencing over standard genetic testing in the amyotrophic lateral sclerosis clinic
Source: Eur J Neurol. 2023 May 28;30(8):2240–9. doi: 10.1111/ene.15855 (PMC10947345; doi:10.1111/ene.15855)
Supplement: Supplementary file 1 — Table S1. [file ENE-30-2240-s001.doc]

**Supplementary Table 1: Co-occuring variants in this study**

| Individual  Number | Variant 1 | Variant 2 |
| --- | --- | --- |
| *1* | C9orf72 HRE | CFAP410 V58L |
| *2* | C9orf72 HRE | CCNF V714M |
| *3* | C9orf72 HRE | OPTN M98K |
| *4* | C9orf72 HRE | KIF5A P986L |
| *5* | FUS R514S | TBK1 V464A |
| *6* | NEK1 S1008X | CCNF V714M |
| *7* | ANXA11 G38R | TBK1 V464A |
| *8* | CFAP410 V58L | CCNF V714M |
| *9* | KIF5A P986L | TBK1 V464A |
| *PLS 1* | PFN E117G | TBK1 V464A |
